# Detection of North American West Nile virus in animal tissue by a reverse transcription-nested polymerase chain reaction assay.

**DOI:** 10.3201/eid0704.010425

**Published:** 2001

**Authors:** D J Johnson, E N Ostlund, D D Pedersen, B J Schmitt

**Affiliations:** Animal and Plant Health Inspection Service, U.S. Department of Agriculture, 1800 Dayton Ave., Ames, IA 50010-0844, USA. donna.j.johnson@aphis.usda.gov

## Abstract

A traditional single-stage reverse transcription-polymerase chain reaction (RT-PCR) procedure is effective in determining West Nile (WN) virus in avian tissue and infected cell cultures. However, the procedure lacks the sensitivity to detect WN virus in equine tissue. We describe an RT-nested PCR (RT-nPCR) procedure that identifies the North American strain of WN virus directly in equine and avian tissues.


### Detection of North American West Nile Virus in Animal Tissue by a Reverse Transcription-Nested Polymerase Chain Reaction Assay

**Donna J. Johnson, Eileen N. Ostlund, Douglas D. Pedersen, and Beverly J. Schmitt**
Animal and Plant Health Inspection Service, U. S. Department of Agriculture, Ames, Iowa, USA

A traditional single-stage reverse transcription-polymerase chain reaction (RT-PCR) procedure is effective in determining West Nile (WN) virus in avian tissue and infected cell cultures. However, the procedure lacks the sensitivity to detect WN virus in equine tissue. We describe an RT-nested PCR (RT-nPCR) procedure that identifies the North American strain of WN virus directly in equine and avian tissues.

West Nile (WN) encephalitis is an infectious, noncontagious, arthropod-borne viral disease (1). WN virus belongs to the family *Flaviviridae*, genus *Flavivirus*. Mosquito vectors transmit the virus among bird populations and, incidentally, to susceptible mammalian species, including humans and horses. While infected horses may not exhibit clinical symptoms, fatal neurologic disease sometimes develops. The emergence of WN virus in the northeast United States in 1999 and 2000 caused concern among horse owners and veterinary practitioners (2). Animal exposure to WN virus can be confirmed serologically by using immunoglobulin M (IgM)-capture enzyme-linked immunosorbent assay (MAC-ELISA) and plaque reduction virus neutralization (PRNT) assays (3,4). Detecting WN virus in animal tissues, by isolation in cell culture or polymerase chain reaction (PCR), confirms infection. Cell culture isolation of WN virus from equine brain tissue can be difficult. It is speculated that the virus does not replicate to high titer in equine brains (Johnson, unpub. observation). PCR procedures have been developed to detect the New York strain (NY99) of WN virus (3,5). A traditional single-stage reverse transcription PCR (RT-PCR) can identify WN virus in avian tissue and mosquitoes (3)  However, the single-stage RT-PCR often lacks the sensitivity to identify WN virus in equine brain (Johnson, unpub. observation). We developed a RT-nested PCR (RT-nPCR) to rapidly and accurately detect WN virus in equine brain.

### The Study

Selected primers amplified a portion of the E region of the genome of WN virus NY99, which encodes the envelope protein (GenBank Accession Number AF196835). This region was highly conserved among several WN virus isolates obtained from the United States in 1999, as well as other WN virus strains of the same lineage (5). The Primer Designer 4 computer software program (Scientific and Educational Software, Durham, NC) was used to select primers. First-stage primer sequences, amplifying a 445-bp region, were 1401: 5'-ACCAACTACTGTGGAGTC-3', and 1845: 5'-TTC-CATCTTCACTCTACACT-3'. Nested primers amplifying a 248-bp region were 1485: 5'-GCCTTCATACACACTAAAG-3' and 1732: 5'-CCAATGCTATCACAGACT-3'.

Total RNA was extracted from 50-100 mg of tissue by using Trizol reagent (Life Technologies, Grand Island, NY) according to the manufacturer's instructions. All samples were extracted and tested in duplicate. A WN virus control was prepared by extracting RNA from a 100-µL volume containing 10 50% tissue culture infective dose WN stock virus. Extracted RNA samples were resuspended in 12 µL RNase-free water and denatured at 70°C for 10 min. Two microliters of each denatured RNA sample was added to 48 µL of RT-PCR mixture with the final composition of 10 mM Tris-HCl (pH 8.3), 50 mM KCl, 2.0 mM $MgCl_2$, 0.8 mM deoxynucleoside triphospate (dNTP) pool, 25 units M-MLV RT, 1.25 units RNase inhibitor, 1.25 units AmpliTaq Gold (Applied Biosystems, Foster City, CA), and 37.5 pmol each of the two first-stage primers. Similarly, 2.0 µL of RNase-free water was added to "no-template" controls that were placed between diagnostic samples. Reaction tubes were incubated at 45°C for 45 min and 95°C for 11 min, followed by 35 cycles of 30-sec denaturation at 95°C, 45-sec primer annealing at 55°C, and 60-sec primer extension at 72°C. The final cycle had similar conditions except for a 5-min primer extension period. For the nested reaction, 1.5 µL of the first-stage amplification product was added to a tube containing 48.5 µL of a PCR mixture with a final composition of 10 mM Tris-HCl (pH 8.3), 50 mM KCl, 2.0 mM $MgCl_2$, 0.8 mM dNTP pool, 1.25 units AmpliTaq Gold, and 37.5 pmol each of the nested primers. Reaction tubes were incubated for 11 min at 95°C, followed by 35 cycles of the cycling conditions described for the first stage. All incubation and amplification procedures were performed by using a Perkin-Elmer 9600 PCR system (Applied Biosystems, Foster City, CA). RT-nPCR product was analyzed by agar gel electrophoresis followed by ethidium bromide staining and UV visualization. A 248-bp product indicated WN virus RNA was present in the original sample (Figure 1). Duplicate samples with discrepant results were retested. No-template controls were used to detect possible cross-contamination. PCR products of selected reactions were

Address for correspondence: Donna J. Johnson, National Veterinary Services Laboratories, P.O. Box 844, Ames, IA 50010, USA; fax: 515-663-7348; e-mail: donna.j.johnson@aphis.usda.gov

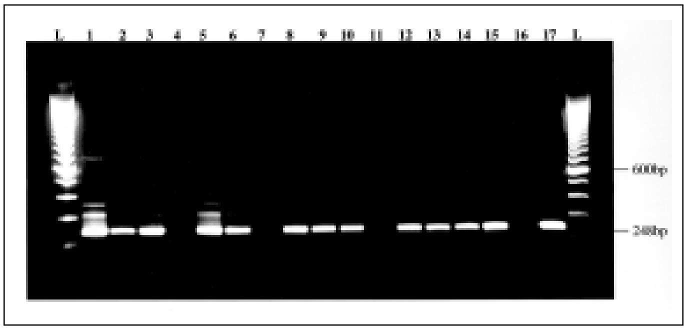

Figure 1. Visualization of reverse transcription-nested polymerase chain reaction product. West Nile virus-positive samples are indicated by a 248-bp band. Lane 1: positive crow brain, NY, 1999. Lane 2: positive crow kidney, NY, 1999. Lane 3: positive Sandhill Crane brain, CT, 1999. Lane 4: negative crow brain. Lane 5-6: positive crow brains, NY, 2000. Lane 7: normal control. Lane 8-10: positive horse brains, NY, 1999. Lane 11: negative horse brain. Lane 12: positive horse brain, RI, 2000. Lane 13: positive horse brain, NJ, 2000. Lane 14: positive horse brain, MA, 2000. Lane 15: positive horse brain, CT, 2000. Lane 16: normal control. Lane 17: NY99 West Nile virus control. L: 100-bp DNA ladder. Band artifacts in lanes 1, 3, 5, and 6 are due to large amount of virus present in those samples.

sequenced and compared with the published sequence of the NY99 strain of WN virus (5, GenBank Accession Number AF196835).

Sensitivity of the RT-nPCR was determined by comparing the endpoint dilution of NY99 WN stock virus detected in Vero cell culture with the endpoint dilution detected by RT-PCR. Tenfold dilutions of virus were prepared. Each dilution was tested in duplicate by cell culture and RT-nPCR. The endpoint dilution in cell culture was $10^{-4.5}$/100 µL. Endpoint dilutions for detection by RT-PCR were $10^{-2.5}$/100 µL after first-stage amplification, and $10^{-8.0}$/100 µL after nested amplification (Figure 2).

Specificity of the RT-nPCR was examined by testing viral RNA extracted from St. Louis encephalitis virus, a closely related flavivirus, as well as bovine viral diarrhea virus and classic swine fever virus, two other *Flaviviridae* family members. Eastern equine encephalitis virus and western equine encephalitis virus, two unrelated North American arboviruses affecting horses, were also tested. The RT-nPCR procedure performed on these samples did not result in observable amplification. Other reference strains of WN virus were not available for testing.

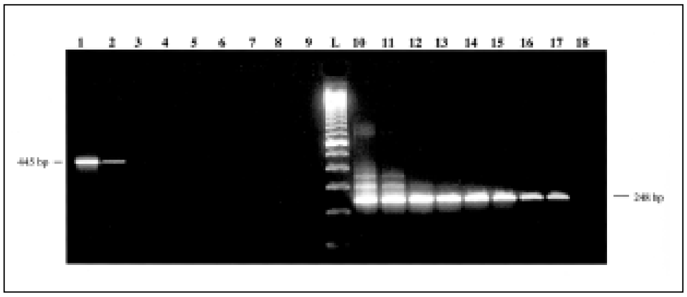

Figure 2. Visual comparison of first stage reverse transcription-polymerase chain reaction (RT-PCR) amplification products with RT-nested (n) PCR amplification products. Lanes 1-9 represent first-stage products of 10-fold dilutions $10^{-1}$ through $10^{-9}$. Lanes 10-18 represent nested amplification products of same dilutions. L: 100-bp DNA ladder.

A total of 128 equine samples were used. Equine brain, blood, and cerebrospinal fluid (CSF) samples collected from suspect WN virus-infected horses were submitted to the National Veterinary Services Laboratories for testing during the 1999 and 2000 outbreak seasons. Diagnostic samples from 31 birds were also tested. All equine and avian brain tissues were tested by RT-nPCR and virus isolation using Vero and rabbit kidney cell cultures. Specimens derived from equine blood samples were also tested serologically, by virus isolation, or both. Five CSF samples from serologically positive horses were tested by RT-nPCR.

Seventy-three equine brains were tested for WN virus by RT-nPCR with 13 yielding positive results (Table). Retrospectively, the first-stage RT-PCR amplification products from the positive samples were examined by agar gel electrophoresis and ethidium bromide staining; two produced a faint band corresponding to the first-stage product of 445 bp (data not shown). The 13 RT-nPCR-positive horses had exhibited typical neurologic signs before death, and sera submitted from all 13 animals were positive for WN virus antibodies by PRNT or MAC-ELISA. WN virus was isolated in cell culture from 10 RT-nPCR-positive horses. Attempts to isolate virus from the remaining three were unsuccessful. The remaining 60 equine brains were negative for WN virus by RT-nPCR and isolation. Sera were available from 15 of the 60 RT-nPCR-negative horses; all 15 were negative for WN virus antibodies. Additional equine samples tested by RT-nPCR were plasma (35 samples), serum (10 samples), buffy coat (5 samples), and CSF (5 samples). Several of these samples were from serologically positive animals; however, all RT-nPCR tests on these samples were negative. Plasma samples were obtained from two ponies experimentally challenged with WN virus; RT-nPCR detected WN virus in plasma from 3 to 7 days postinoculation (dpi) in one pony and 3-6 dpi in the second pony (Table). Virus was isolated from the plasma of one pony at 6 dpi. The ponies were not exhibiting clinical symptoms.

Thirty-one avian brain samples were also tested. Seven of these samples were positive for WN virus by RT-nPCR and virus isolation (Table). The remaining 24 samples were negative by both procedures. Positive RT-nPCR results were also obtained from tissues (e.g., kidney, spleen, and liver) from some positive birds (Figure 1, lane 2).

RT-nPCR amplification products from 6 positive samples collected in 1999 (3 equine, 3 avian) and 12 positive samples

Table. Virus isolation compared with plaque reduction virus neutralization, immunoglobulin M-capture enzyme-linked immunosorbent assay serologic results, or both for West Nile virus samples positive by reverse transcription-nested polymerase chain reaction

| RT-nPCR positive samples | Results | | | | |
|---|---|---|---|---|---|
| | No. tested | VI positive | VI negative | Sero-positive | Sero-negative |
| Equine brain | 13 | 10 | 3 | 13 | 0 |
| Equine plasma | 8[a] | 1 | 7 | 0 | 8[b] |
| Avian brain | 7 | 7 | 0 | NA[c] | NA |

RT-nPCR = reverse transcription-nested polymerase chain reaction; VI = virus isolation; NA = not applicable.
[a]Multiple plasma samples collected from two experimentally challenged ponies were RT-nPCR-positive for West Nile virus between 3 and 7 days after inoculation.
[b]West Nile virus antibodies were not detected in either pony until beyond the time the virus was detected by RT-nPCR.
[c]No avian serum was available for testing.

from 2000 (10 equine and 2 avian) were sequenced and compared with the published NY99 WN virus sequence (5). Within the 248-bp amplified region, two horse samples, one each from 1999 and 2000, had one base change; the remaining 16 samples were identical to NY99 WN virus.

### Conclusions

The RT-nPCR proved to be a rapid and reliable method for detecting WN virus in equine as well as avian tissues. While isolation of WN virus from the 10 equine isolates required up to two passages in cell culture and 7-14 days to complete, WN virus was confirmed in tissue by RT-nPCR in <24 hours.

End-point dilution tests determined the RT-nPCR procedure to be 1,000-fold more sensitive than cell culture for detecting WN virus. Additionally, nested amplification increased the sensitivity of the RT-PCR at least 100,000-fold over first-stage amplifiction (Figure 2). Comparison of virus isolation and RT-nPCR results further demonstrated the increased sensitivity of RT-nPCR. All RT-nPCR-positive brain samples were confirmed by virus isolation except three equine brain tissues; WN virus infection in those animals was confirmed serologically. Had the procedure consisted of only first-stage amplification, only two of the positive horses would have been correctly identified.

Specificity of the RT-nPCR test was confirmed by sequence analysis of the amplified products of 18 positive samples and by absence of amplification of St. Louis encephalitis, bovine viral diarrhea, classic swine fever, eastern equine encephalitis, and western equine encephalitis viruses. Complete agreement between negative RT-nPCR tests on brain tissues and all other WN virus diagnostic tests performed further demonstrates the specificity of the assay in identifying animals not infected with WN virus.

Equine blood-derived samples and CSF samples from clinically ill animals were not reliable for determining the presence of WN virus by either RT-nPCR or isolation. Limited information from two experimentally challenged ponies indicated a several-day viremic phase early after infection, before WN virus antibody was detectable. Taken together, our data suggest that the viremic phase of infection occurs before clinical symptoms develop. Therefore, the RT-nPCR would not be expected to detect WN virus in blood of horses exhibiting clinical signs of WN virus encephalitis. Additionally, while WN virus-specific IgM antibodies may be detected in equine CSF during clinical illness, CSF samples do not appear to have diagnostic value for detecting WN virus RNA in horses.

As with all nested PCR procedures, additional manipulation of reaction tubes can lead to cross-contamination, corrupting the test (6). Confidence in the procedure may be increased by testing duplicate samples and including multiple controls.

### Acknowledgments

We thank Kathryn Moser and Kevin Lake for technical assistance with sample processing and diagnostic testing.

Ms. Johnson is a microbiologist in the Diagnostic Virology Laboratory of the National Veterinary Services Laboratories. Her major focus is equine and ovine viral diseases.

### References

1. Monath TP, Heinz FX. Flaviviruses. In: Fields BN, Knipe DM, Howley PM, editors. Fields virology. 3rd ed. Philadelphia: Lippincott-Raven Publishers; 1996. p. 961-1034.
2. Anderson JF, Andreadis TG, Vossbrinck CR, Tirrell S, Wakem EM, French RA, et al. Isolation of West Nile virus from mosquitoes, crows, and a Cooper's Hawk in Connecticut. Science 1999;286:2331-3.
3. Lanciotti RS, Kerst AJ, Nasci RS, Godsey MS, Mitchell CJ, Savage HM, et al. Rapid detection of West Nile virus from human clinical specimens, field-collected mosquitoes, and avian samples by a TaqMan reverse transcriptase-PCR assay. J Clin Microbiol 2000;38:4066-71.
4. Tardei G, Ruta S, Chitu V, Rossi C, Tsai TF, Cernescu C. Evaluation of immunoglobulin M (IgM) and IgG enzyme immunoassays in serologic diagnosis of West Nile virus infection. J Clin Microbiol 2000;38:2232-9.
5. Lanciotti RS, Roehrig JT, Deubel V, Smith J, Parker M, Steele K, et al. Origin of the West Nile virus responsible for an outbreak of encephalitis in the northeastern United States. Science 1999;286:2333-7.
6. Roux KH. Optimization and troubleshooting in PCR. In: Dieffenbach CW, Dveksler GS, editors. PCR primer, a laboratory manual. Plainview (NY): Cold Spring Harbor Laboratory Press; 1995. p. 53-62.
